# Retraction: MicroRNA-96-5p promotes proliferation, invasion and EMT of oral carcinoma cells by directly targeting FOXF2

**DOI:** 10.1242/bio.059527

**Published:** 2022-07-28

**Authors:** Haiyan Wang, Ning Ma, Wenyue Li, Zuomin Wang

The journal has retracted *Biology Open* (2020) **9**, bio049478 (doi:10.1242/bio.049478) because of unreliable data and author fraud. This notice updates and replaces the previous Retraction and Expression of Concern notices.

Cell images in this paper were reported to be duplicated in two other articles by a commenter on PubPeer. Our analysis found that many of the western blots and several cell images were unexpectedly very similar to those in other articles, where they are presented for completely different cell lines and different experimental conditions. There is no overlap in authorship of the three papers and several other flags indicative of outsourced ‘papermill’ papers were also noted. The journal therefore retracted the article. The corresponding author listed, Zuomin Wang, contacted the journal after seeing news coverage on the retraction to say that she had no knowledge of this paper.

An investigation was carried out by a team at the corresponding author's institute, Beijing Chaoyang Hospital, Capital Medical University. The team report that:

1. Without their knowledge, Wenyue Li and Zuomin Wang were added as authors of the paper by Haiyan Wang using fake email addresses that were used for submission and correspondence with Biology Open.
2. The study was conducted in Qingdao Municipal Hospital and was approved by the ethics review committee of Qingdao Municipal Hospital. No application was received by the ethics review committee of Beijing Chaoyang Hospital. Haiyan Wang participated in this study as an independent investigator without authorization from Beijing Chaoyang Hospital.
3. Haiyan Wang and Ning Ma collected tissue samples together at Qingdao Municipal Hospital, then Ning Ma outsourced the analysis to a commercial company. The investigation team's review of the original data and images received by Ning Ma showed several signs of forgery, including inconsistency between sample sizes and incomplete data, etc.

The BCH investigation team concluded that: ‘the practices of preparation and submission of this study for publication clearly violate academic integrity and ethics. We also strongly question the reliability of the data reported.’

The first author, Haiyan Wang, has contacted the journal admitting supplying fake email addresses and ORCiDs, which prevented Zuomin Wang and Wenyue Li from receiving any correspondence. Biology Open requires ORCiDs and/or non-institutional emails for all authors and apologises to Wenyue Li and Zuomin Wang that, despite our checks on this paper, it was published fraudulently in their names.

